# *Morinda citrifolia* L. Leaf Extract Protects against Cerebral Ischemia and Osteoporosis in an *In Vivo* Experimental Model of Menopause

**DOI:** 10.1155/2018/1039364

**Published:** 2018-03-25

**Authors:** Jintanaporn Wattanathorn, Cholathip Thipkaew, Wipawee Thukham-mee, Supaporn Muchimapura, Panakaporn Wannanon, Terdthai Tong-un

**Affiliations:** ^1^Integrative Complementary Alternative Medicine Research and Development Center, Khon Kaen University, Khon Kaen 40002, Thailand; ^2^Department of Physiology, Faculty of Medicine, Khon Kaen University, Khon Kaen 40002, Thailand

## Abstract

We aimed to determine the protective effects against cerebral ischemia and osteoporosis of *Morinda citrifolia* extract in experimental menopause. The neuroprotective effect was assessed by giving *M. citrifolia* leaf extract at doses of 2, 10, and 50 mg/kg BW to the bilateral ovariectomized (OVX) rats for 7 days. Then, they were occluded in the right middle cerebral artery (MCAO) for 90 minutes. The neurological score, brain infarction volume, oxidative stress status, and ERK1/2 and eNOS activities were assessed 24 hours later. *M. citrifolia* improved neurological score, brain infarction, and brain oxidative stress status in the cortex of OVX rats plus the MCAO. No changes in ERK 1/2 signal pathway and NOS expression were observed in this area. Our data suggested that the neuroprotective effect of the extract might occur partly via the improvement of oxidative stress status in the cortex. The antiosteoporotic effect in OVX rats was also assessed after an 84-day intervention of *M. citrifolia*. The serum levels of calcium, osteocalcin, and alkaline phosphatase and osteoblast density in the tibia were increased, but the density of osteoclast was decreased in OVX rats which received the extract. Therefore, the current data suggested that the extract possessed antiosteoporotic effect by increasing bone formation but decreasing bone resorption.

## 1. Introduction

The number of menopausal women is continuously increasing accompanied with the rising of the aging population. During this period, the risks of stroke [1] and osteoporosis are increased [2]. It has been reported that the disturbance of oxidative stress regulation plays the crucial role in the pathophysiology of stroke and osteoporosis in menopausal women [3]. Therefore, antioxidant has been considered as one of the targets for both prophylactic and therapeutic intervention.

During the last decade, accumulative lines of evidence have demonstrated that substances possessing antioxidant effect are the promising therapeutic agent against stroke [4] and osteoporosis in ovariectomized (OVX) rat [5]. Based on these pieces of information, the preventive effect against stroke and osteoporosis of a medicinal plant possessing antioxidant activity has been considered.

The leaf of *Morinda citrifolia* L. (Noni) or Yor in Thai has been widely consumed in Thai cuisine such as curry and hor mok or steamed fish with curry paste in a banana leaf cup. It has been reported that *M. citrifolia* leaf is rich in numerous nutrients and minerals including calcium. In Thailand and many countries in both Southeast Asia and Polynesia, the leaf of *M. citrifolia* can be used not only as food but also as medicine. In addition, according to Thai traditional folklore, *M. citrifolia* leaf has been used for treating muscle pain, inflammation, and hypertension. The leaf extract also decreases triglyceride, cholesterol, low-density lipoprotein cholesterol (LDL-C), and atherogenic index in an animal model of dyslipidemia induced by high-fat diet [6]. In addition, the previous study also demonstrated the antioxidant effect of *M. citrifolia* leaf [7]. Due to the biological effects of *M. citrifolia* mentioned earlier, we hypothesized that the extract of the leaves of *M. citrifolia* should decrease the risks of both stroke and osteoporosis in menopausal condition. Since no data are available, we aimed to determine the neuroprotective and antiosteoporotic effects of *M. citrifolia* leaf extract in ovariectomized rats, an animal model of menopausal condition.

## 2. Materials and Method

### 2.1. Plant Material and Extract Preparation

**Figure 1:**
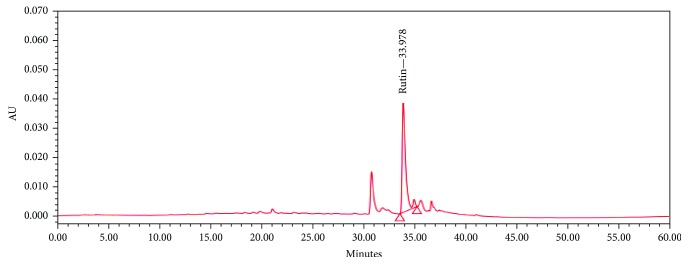
Fingerprint chromatogram of *M. citrifolia* leaf extract.

The leaves of *M. citrifolia* are collected from an organic farm at Nakorn Nayok Province, Thailand, during February 2015. The plant was identified by Mr. Winai Somprasong, agricultural scientist in Botany and Plant Herbarium Research Group, Plant Varieties Protection Division. After the authentication, they were cleaned with tap water and rinsed with distilled water in order to remove the contaminants and debris. Then, they were dried in an oven at 40°C, ground into powder with a grinder, and extracted with water using maceration technique for 72 hours (1 : 2 *w*/*v*). The extract was filtered and lyophilized using freeze dryer to give a powder of an aqueous extract of *M. citrifolia* leaves (yield 7.14%). The extract contained total phenolic compounds and rutin at the concentrations of 374.55 ± 0.47 mg of gallic acid equivalent (GAE)/g extract and 5.75 ± 0.86 mg/g extract, respectively, as shown in Figure 1.

### 2.2. Experimental Animals

Healthy female Wistar rats (180–200 g) from the National Laboratory Animal Center, Salaya, Nakorn Pathom, were used as experimental animals. They were randomly housed 5 per cage, maintained in 12 : 12 light : dark cycle, and given access to food and water ad libitum. The experiments were strictly performed in accordance with the internationally accepted principles for laboratory use and care of the European Community (EEC Directive of 1986; 86/609/EEC). The experiment protocols were approved by the Institutional Animal Care and Unit Committee, Khon Kaen University, Thailand (Record number AEKKU29/2558). All operations were performed under the pentobarbital sodium anesthesia in order to minimize animal suffering.

### 2.3. Experimental Procedures

#### 2.3.1. Neuroprotective Effect of *M. citrifolia* in Experimental Menopause

After 1 week of acclimatization, female Wistar rats weighing 180–200 g were induced bilateral ovariectomy to mimic the menopausal condition. To assure the situation of estrogen reduction, the vaginal epithelium was smeared, and only the rats which showed scant amount of leukocytes and mucus and a few epithelial cells were recruited for further study. They were randomly divided into various groups as follows:

1. Ovariectomized rat (OVX) + sham operation: all OVX rats in this group were exposed to sham operation and received vehicle (distilled water).
2. OVX rats + MCAO: the OVX rats in this group were orally given vehicle for 7 days and subjected to the ischemic-reperfusion injury by inducing a temporary occlusion of right MCA (MCAO) for 90 minutes.
3. OVX + MCAO plus genistein: all OVX rats in this group were orally given genistein, a well-known isoflavonoid which produces the advantage on menopause-related problems including brain function and osteoporosis (15 mg/kg BW), for 7 days, and subjected to ischemic-reperfusion injury at MCA as mentioned above.
4. OVX + MCAO + MC 2: the OVX rats were administered *M. citrifolia* at a dose of 2 mg/kg BW via oral route for 7 days and subjected to MCAO as mentioned above.
5. OVX + MCAO + MC 10: the OVX rats were administered *M. citrifolia* at a dose of 10 mg/kg BW via oral route for 7 days and subjected to MCAO as mentioned above.
6. OVX + MCAO + MC 50: the OVX rats were administered *M. citrifolia* at a dose of 50 mg/kg BW via oral route for 7 days and subjected to MCAO as mentioned above.

At the end of the experiment, the neurological score for all animals was determined at 24 hours after MCAO; the brains were isolated and brain infarction volume, oxidative stress status, extracellular signal-regulated protein kinases 1 and 2 (ERK1/2) activity, and endothelial nitric oxide synthase (eNOS) activity were determined.

#### 2.3.2. Antiosteoporotic Effect of *M. citrifolia* in Experimental Menopause

After 1 week of acclimatization period, female Wistar rats (180–200 g) were randomly divided into various groups described as follows:

1. Sham operation: all rats were exposed to sham operation and received vehicle (distilled water) once daily for 84 days.
2. OVX + vehicle: all animals in this group were induced bilateral ovariectomy and received vehicle once daily for 84 days.
3. OVX + genistein: all rats were induced bilateral ovariectomy and received genistein (15 mg/kg), once daily for 84 days.
4. OVX + MC: all rats in these groups were induced bilateral ovariectomy and orally treated with *M. citrifolia* extract at doses of 2, 10, and 50 mg/kg BW, respectively, once daily for 84 days.

At the end of the study, serum bone markers including osteocalcin, calcium, and alkaline phosphatase of all the rats were determined. Tibia bone was isolated and osteoblast and osteoclast densities were determined.

### 2.4. Ovariectomy and Ischemic-Reperfusion Injury Induction

After 2 weeks of acclimatization, the female rats were anesthetized with pentobarbital sodium (50 mg/kg, via intraperitoneal (i.p.) route), and their ovaries were removed bilaterally. All rats in the sham-operated group were anesthetized, laparotomized, and sutured without removing their ovaries. After the ovariectomy, the animals were orally given vehicle or various doses of *M. citrifolia* extract ranging from 2, 10, and 50 mg/kg BW for 7 days. Then, they were induced ischemic reperfusion injury by inserting a 4-0 nylon filament with rounded tip into the right middle cerebral artery (MCA) to produce the occlusion for 90 minutes. Reperfusion was established again when the filament was withdrawn.

### 2.5. Assessment of Neurological Score

A neurological score was determined according to the method of Garcia [7] 24ss hours after the occlusion. A rating scale ranging from 0 to 3 was used for grading the deficit in 6 different aspects including spontaneous activity, symmetry in the movement of the 4 limbs, forepaw outstretching, climbing, body proprioception, and response to vibrissal touch. According to this method, the score in healthy rats would be 18.

### 2.6. Evaluation of Brain Infarction Volume

The measurement of brain infarct volume was carried out by using 2,3,5-triphenyl tetrazolium chloride (TTC) staining. The animals were sacrificed 24 hours after ischemic-reperfusion injury (IR). The brains were removed and cut as coronal sections at 2 mm thick (from anterior, 3.5 mm to anterior, 13.5 mm from bregma). Then, a 30-minute immersion in 2% TTC at 37°C was performed. The TTC-stained brain sections were photographed, and the infarction area was determined by measuring the white area of the brain section with computer software (Image J 1.4 V). All assessments were performed by an investigator blinded to the type of interventions.

### 2.7. Homogenate Preparation

At the end of the experiment, the preparation of homogenate containing the right cortex and subcortex was performed in 1 ml of 0.1 M phosphate buffer, pH 7.4. Then, the brain homogenate was adjusted to 10% *w*/*v* and centrifuged with microcentrifuge (SIG 1-15PK) at 3000*g*, 4°C for 15 minutes. The supernatant was harvested and used for further biochemical assessments.

### 2.8. Biochemical Analyses

#### 2.8.1. Malondialdehyde (MDA) Level Assessment

The assessment of malondialdehyde (MDA) level in the cortex and subcortex was carried out by using thiobarbituric acid-reacting substance (TBARS) assay. In brief, an aliquot of sample at 100 *μ*l was mixed with the solution containing 100 *μ*l of 8.1% (*w*/*v*) sodium dodecyl sulphate (Sigma-Aldrich), 750 *μ*l of 20% (*v*/*v*) acetic acid (Sigma-Aldrich) (pH 3.5), and 750 *μ*l of 0.8% thiobarbituric acid (TBA) (Sigma-Aldrich). The solution was heated in a water bath at 95°C for one hour and cooled immediately under running tap water. Then, 500 *μ*l of chilled water and 2500 *μ*l of butanol and pyridine (Sigma-Aldrich) (15 : 1 *v*/*v*) were added into each tube and mixed thoroughly with vortex (Vortex-Genie 2). Then, the solution was subjected to an 800*g* centrifugation for 20 minutes. The upper layer was harvested, and the optical density was measured at 532 nm with spectrophotometer (GENESYS 20). Various concentrations of 1,3,3-tetraethoxypropane (TEP) (Sigma-Aldrich) were used as standard [8]. The level of MDA was expressed as nmol/mg·protein.

#### 2.8.2. Determination of Scavenging Enzyme Activities

Superoxide dismutase (SOD) activity was determined based on the inhibitory effect of SOD on the reduction of nitroblue tetrazolium (NBT) by the superoxide anion generated by the system xanthine/xanthine oxidase as previously described elsewhere [9]. Absorbance at 550 nm was measured using a spectrometer (UV-1601, Shimadzu), and SOD activity was presented as units per milligram of protein (U/mg protein). One unit of enzyme activity was defined as the quantity of SOD required to inhibit the rate of reduction of cytochrome by 50%. Catalase (CAT) activity was determined based on the disappearance of H_2_O_2_ in the presence of brain homogenate at 490 nm [10]. The activity of CAT was expressed as *μ*mol H_2_O_2_/min/mg protein. The determination of glutathione peroxidase (GPx) was performed using t-butylhydroperoxide as substrate [11]. The activity was expressed as U/mg protein. One unit of the enzyme was defined as micromole (*μ*mol) of reduced nicotinamide adenine dinucleotide phosphate (NADPH) oxidized per minute.

#### 2.8.3. Assessments of Serum Calcium, Alkaline Phosphatase, and Osteocalcin

The animals were deeply anesthetized with pentobarbital sodium (50 mg/kg, i.p.), and blood was collected from the abdominal thoracic aorta and centrifuged at 3000 rpm for 10 minutes. Then, the serum was isolated and kept at −20°C for the determination of serum calcium, alkaline phosphatase, and osteocalcin. The serum osteocalcin level was determined by using Rat Gla-Osteocalcin High Sensitive EIA Kit (Takara Bio Inc. Company, Japan), whereas serum calcium and alkaline phosphatase were determined using standard laboratory method.

#### 2.8.4. Assessment of ERK1/2 Activity

The cortex was removed and prepared as brain homogenate, and ERK1/2 activity was determined by using a commercial ELISA kit according to the instruction brochure. In brief, 50 *μ*l of all standards and samples was added to appropriate wells and mixed with 50 *μ*l of antibody cocktail (the combination of 3 ml of 10X ERK1/2 (total) capture antibody and 3 ml of 10X ERK1/2 (total) detector antibody). Then, the plate was sealed and incubated at room temperature on a plate shaker (400 rounds per minute) for 1 hour. After the incubation, the wells were washed 3 times with 350 *μ*l 1X wash buffer, and liquid was removed. Total aliquot of TMB substrate at the volume of 100 *μ*l was added into the wells and incubated in a dark condition on a 400 rpm plate shaker for 15 minutes. Then, the stop solution at the volume of 100 *μ*l was added and mixed thoroughly, and the absorbance at 450 nm was recorded.

#### 2.8.5. Assessment of Nitric Oxide Synthase (NOS) Activity in the Cerebral Cortex

The activity of nitric oxide synthase (NOS) in the cerebral cortex was assessed using a commercial ELISA Kit (CUSABIO). The assessment was performed according to the instruction brochure. In brief, 50 *μ*l of all standards and samples was added to the appropriate wells and mixed with 50 *μ*l of antibody cocktail (the combination of rat nitric oxide synthase lyophilized capture antibody and 10X nitric oxide synthase 1 detector antibody in antibody diluent 4BI). Then, the plate was sealed and incubated at room temperature on a 400 rpm plate shaker for 1 hour. After the incubation, each well was washed 3 times with 350 *μ*l 1X wash buffer, and liquid was removed. Then, 100 *μ*l of TMB substrate was added to each well and incubated in the dark condition on a 400 rpm plate shaker for 15 minutes. At the end of the incubation period, 100 *μ*l of stop solution was added to each well, mixed, and subjected to shaking on a plate shaker for 1 minute. Then, the optical density at 450 nm was recorded.

### 2.9. Determination of Osteoblast and Osteoclast Densities

The left tibia was removed, cleaned from the adhering muscles, and fixed with PLP fixative (2% paraformaldehyde containing 0.075 M lysine and 0.01 M sodium periodate solution, pH 7.4, stored at 4°C) at 4°C for 24 hours. The tibia bone tissue was dehydrated in a graded series of alcohol and embedded in paraffin wax. The upper end of the tibia was sectioned (5 *μ*m thickness) longitudinally on a rotary microtome and processed for hematoxylin and eosin staining. The densities of osteoblast and osteoclast cells were measured [5].

### 2.10. Statistical Analysis

Data were expressed as means ± SEM and analyzed statistically by SPSS and one-way ANOVA, followed by the post hoc (Tukey) test. The results were considered statistically significant at *p* < 0.05.

## 3. Results

### 3.1. Effect of *M. citrifolia* Extract on Neurological Score and Brain Infarction Volume

Table 1 demonstrated that sham operation produced no changes on the parameters of neurological score including spontaneous activity, symmetry in the movement of the 4 limbs, forepaw outstretching, climbing, body proprioception, and response to vibrissal touch. Ovariectomized (OVX) rats which received ischemic-reperfusion injury at the right middle cerebral artery (MCAO) decreased symmetry in the movement of the 4 limbs of the neurological score (*p* value < 0.05, compared to OVX + sham operation group). Genistein increased body proprioception (*p* value < 0.05, compared to OVX + MCAO group).

*M. citrifolia* extract at doses of 2 and 50 mg/kg BW increased body proprioception and response to vibrissal touch (all *p* value < 0.01; *p* value < 0.001 and 0.01; *p* value < 0.05 and 0.01; compared to OVX rat + MCAO group). OVX rats which received *M. citrifolia* extract at dose of 10 mg/kg improved only body proprioception (*p* value < 0.01, compared to OVX rat + MCAO group).

**Figure 2:**
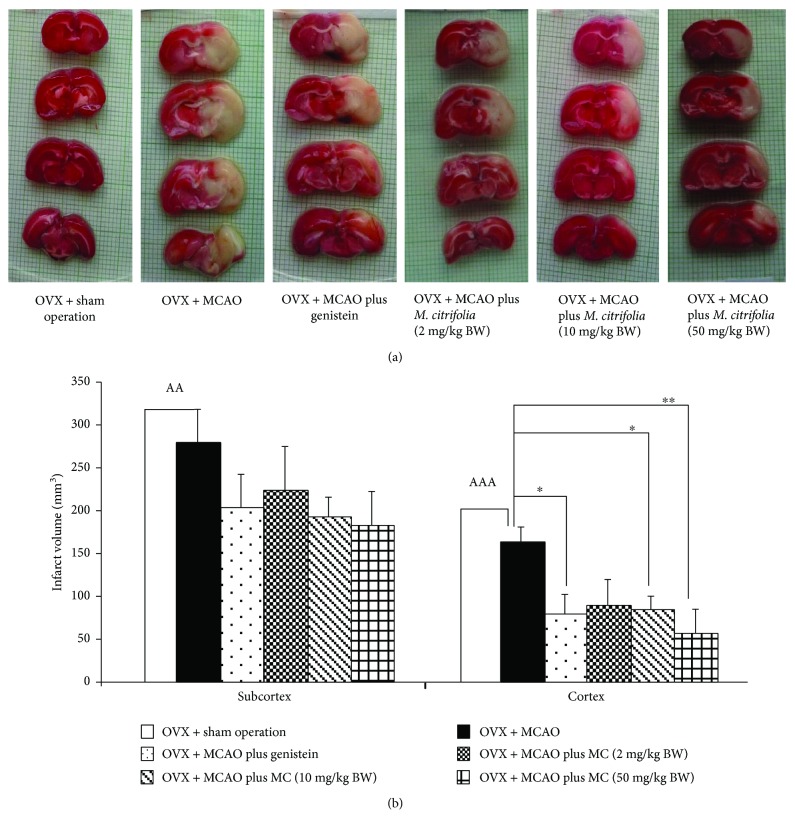
Effect of *M. citrifolia* leaf extract on brain infarction volume which was assessed by using 2,3,5-triphenyl tetrazolium chloride (TTC) staining (*N* = 6/group). Photograph of the (a) brain infarction area; (b) brain infarction volume. ^AA^*p* value < 0.01 and ^AAA^*p* value < 0.001, compared with OVX + sham operation. ^∗^*p* value < 0.05 and ^∗∗^*p* value < 0.01, compared with OVX + MCAO.

The effect of *M. citrifolia* extract on brain infarction volume was also determined, and the results were shown in Figure 2. OVX rats which were subjected to ischemic-reperfusion injury by transient occlusion at the middle cerebral artery (MCAO) increased infarction volume in both the cortex and subcortex (*p* value < 0. 001 and 0.01, respectively, compared to OVX rat + sham operation group). Genistein together with the medium and high doses of *M. citrifolia* extract used in this study significantly decreased brain infarction in the cortex of OVX rats which were subjected to MCAO (*p* value < 0.05, 0.05, and 0.01, respectively, compared to OVX rat + MCAO group).

### 3.2. Effect of *M. citrifolia* Extract on Brain Oxidative Stress Status

It was found that OVX rats which were subjected to MCAO and received vehicle showed the elevation of MDA level in the cerebral cortex (*p* value < 0.001, compared to OVX rat + sham operation group), while no changes in antioxidant activities of the main scavenger enzymes including SOD, CAT, and GPx in the cortex were observed as shown in Table 2. Only OVX rats which were subjected to MCAO and received the extract at a dose of 50 mg/kg BW showed the reduction of MDA level (*p* value < 0.05, compared to OVX rat + MCAO group).

### 3.3. Effect of *M. citrifolia* Extract on ERK1/2 Activity in the Cerebral Cortex

**Figure 3:**
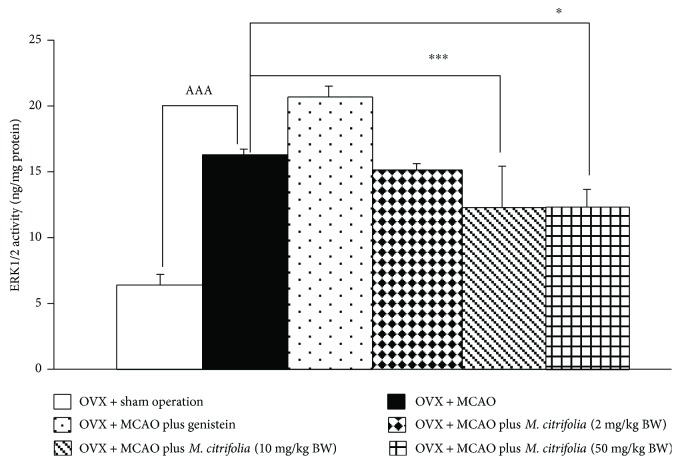
Effect of *M. citrifolia* on the extracellular signal-regulated kinase 1/2 (ERK1/2) activity in the cortex (*N* = 6/group). ^AAA^*p* value < 0.001, compared to OVX + sham operation group. ^∗^*p* value < 0.05 and ^∗∗∗^*p* value < 0.001, compared to OVX + MCAO group.

Since *M. citrifolia* significantly decreased the brain infarction volume, the possible underlying mechanisms were investigated. In addition to oxidative stress status, other factors such as extracellular signal-regulated kinase 1/2 (ERK1/2), one of the best-characterized members of the mitogen-activated protein kinase (MAPK) family, which plays the crucial role in the survival of neuron [12], were also investigated, and data were shown in Figure 3. The results showed that OVX rats which were subjected to MCAO and received vehicle increased ERK1/2 (*p* value < 0.001, compared to OVX rat + sham group). Genistein and all doses of extract failed to modify the expression of ERK1/2 in the cortex.

### 3.4. Effect of *M. citrifolia* on the Activity of Nitric Oxide Synthase

**Figure 4:**
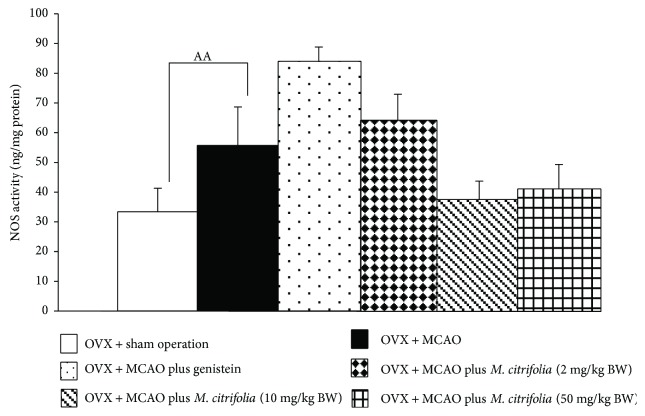
Effect of *M. citrifolia* on the activity of nitric oxide synthase (NOS) in the cortex (*N* = 6/group). ^AA^*p* value < 0.01, compared to OVX + sham operation group.

Based on the crucial role of nitric oxide on brain damage following ischemic stroke [13], we also explored the effect of *M. citrifolia* on the activity of nitric oxide synthase (NOS), a key enzyme playing an important role in nitric oxide (NO) synthesis.  Figure 4 demonstrated that OVX rats which were subjected to MCAO and received vehicle significantly increased NOS activity in the cerebral cortex (*p* value < 0.01, compared to OVX + sham operation group). OVX rats which were subjected to MCAO and received either genistein or all doses of *M. citrifolia* failed to improve this change.

### 3.5. Effect of *M. citrifolia* on the Density of Osteoblast and Osteoclast Densities and on the Bone Formation Markers

**Figure 5:**
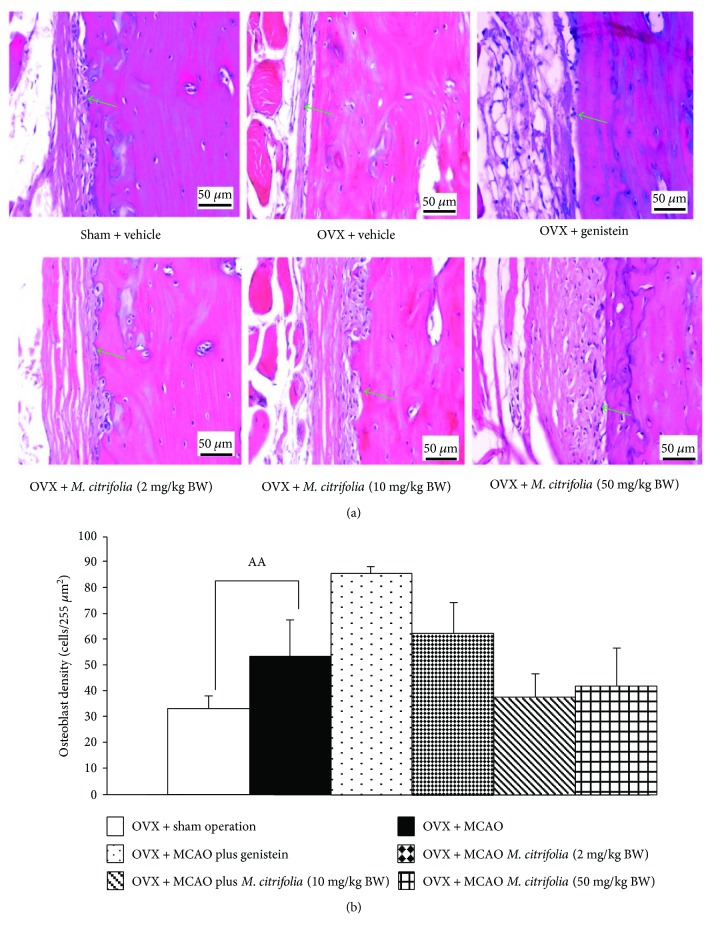
Effect of *M. citrifolia* leaf extract on osteoblast density in the tibia of OVX rats. Photograph of (a) osteoblast in the tibia; (b) osteoblast density (*N* = 6/group). ^AA^*p* value < 0.01, compared to sham operation group. ^∗∗^*p* value < 0.01, compared to OVX group.

**Figure 6:**
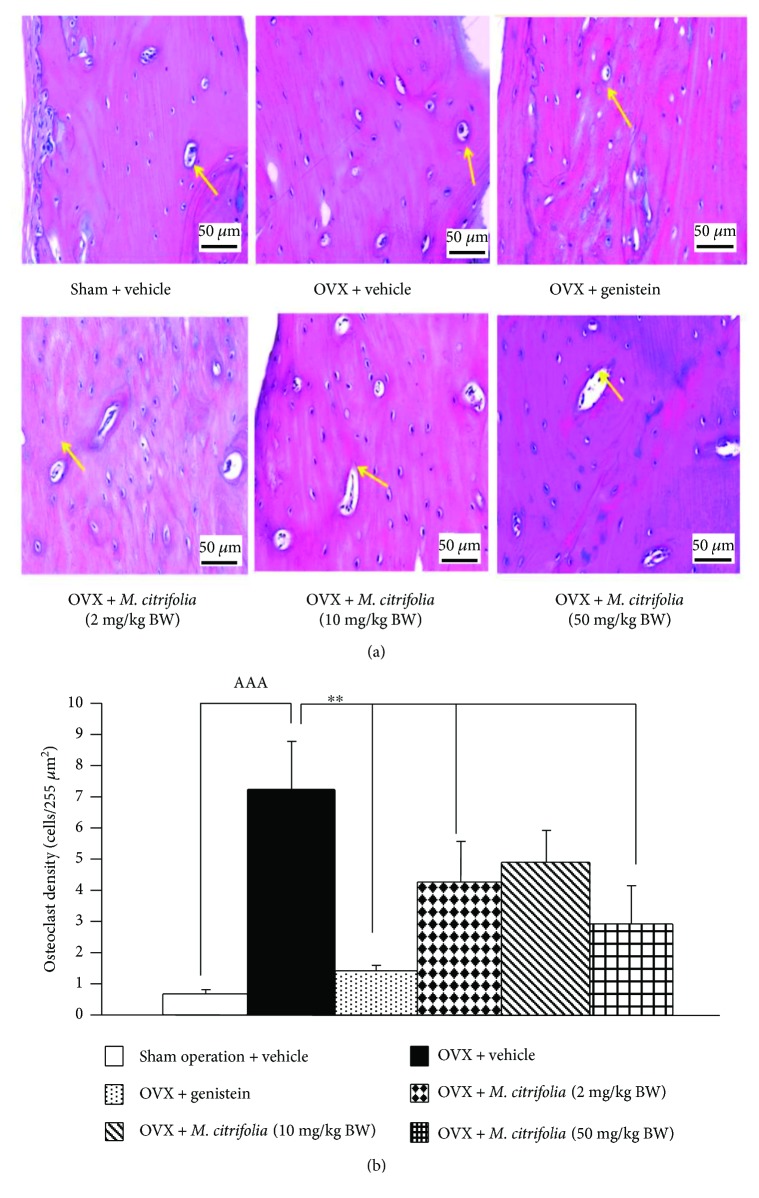
Effect of *M. citrifolia* leaf extract on osteoclast density in the tibia of OVX rats. Photograph of (a) osteoclast in the tibia; (b) osteoclast density (*N* = 6/group). ^AAA^*p* value < 0.001, compared to sham operation group. ^∗∗^*p* value < 0.01, compared to OVX group.

**Figure 7:**
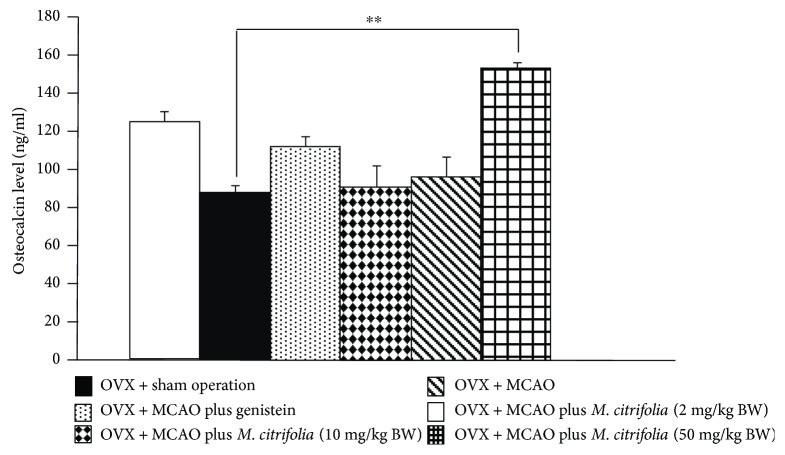
Effect of *M. citrifolia* leaf extract on serum osteocalcin of OVX rats (*N* = 6/group). ^∗∗^*p* value < 0.01, compared to OVX group.

**Figure 8:**
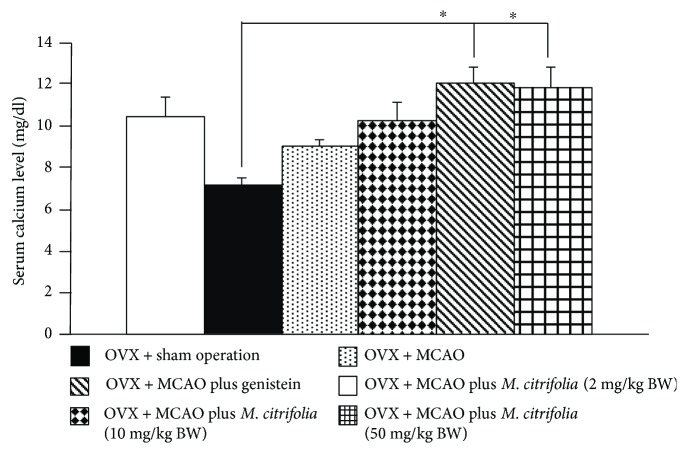
Effect of *M. citrifolia* leaf extract on serum calcium of OVX rats (*N* = 6/group). ^∗^*p* value < 0.05, compared to OVX group.

**Figure 9:**
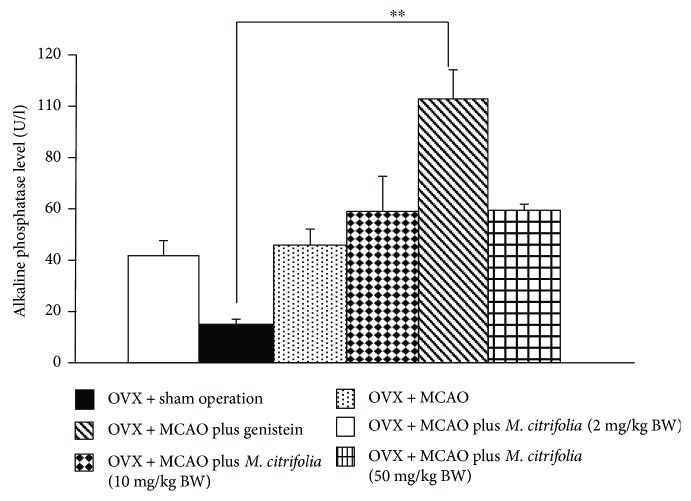
Effect of *M. citrifolia* leaf extract on serum alkaline phosphatase of OVX rats (*N* = 6/group). ^∗∗^*p* value < 0.01, compared to OVX group.

Figures 5 and 6 showed the effect of *M. citrifolia* on the densities of osteoblast and osteoclast in the tibia bone of OVX rats. OVX rats which received vehicle significantly decreased osteoblast density (*p* value < 0.01, compared to sham operation group) but increased osteoclast density (*p* value < 0.001, compared to sham operation group) in the tibia. It was found that OVX rats which received genistein failed to increase osteoblast density but significantly decreased osteoclast density (*p* value < 0.01, compared to OVX group) in the tibia. Interestingly, the reduction of osteoblast density in OVX rats which received MCAO was attenuated by both medium and high doses of *M. citrifolia* leaf extract (all *p* value < 0.05, compared to OVX group). In addition, the increase in osteoclast density in OVX rats which received MCAO was also attenuated by *M. citrifolia* leaf extract at doses of 2 and 50 mg/kg BW (*p* value < 0.05 and 0.01, respectively, compared to OVX group). Moreover, the serum osteocalcin, alkaline phosphatase, and calcium level were also investigated, and data were shown in Figures 7–9. OVX rats which received vehicle failed to show the significant changes of all parameters just mentioned. No significant modulation effect of genistein on serum osteocalcin, alkaline phosphatase, and calcium level in OVX rats was observed. OVX rats which received low dose of extract increased only the serum alkaline phosphatase (*p* value < 0.05, compared to OVX group). Medium dose of the extract significantly increased both serum alkaline phosphatase and serum calcium level (*p* value < 0.001 and 0.01, respectively, compared to OVX group), whereas the high dose of extract produced the significant elevations of serum osteocalcin, alkaline phosphatase, and serum calcium levels (*p* value < 0.01, 0.05, and 0.01, respectively, compared to OVX group).

## 4. Discussion

The current results revealed that the extract of *M. citrifolia* leaves produced the improvement in neurological score and infarction size in the cerebral cortex of OVX rats with MCAO. Meanwhile, *M. citrifolia* also decreased MDA level which indicated the improvement of oxidative stress status in the mentioned area. No significant changes of ERK1/2 signal transduction and NOS activity in the cortex were observed. Interestingly, the increases in bone formation markers including serum levels of osteocalcin, calcium, and alkaline phosphatase were also revealed. The osteoblast density was also increased, but the osteoclast density was decreased following the treatment with *M. citrifolia* extract.

Cerebral ischemia can induce tissue hypoxia, cellular dysfunction, and cell death. The treatment goal is to restore vascular supply to an organ temporarily deprived of blood flow. However, it often paradoxically results in injury of the affected tissue bed [14, 15] or ischemic-reperfusion injury which in turn induces a severe clinical problem with high mortality and morbidity. The underlying mechanism of cerebral ischemic-reperfusion injury is very complex and involves multifactors. Oxidative stress and free radicals have been reported to contribute crucial roles in the cascade pathway of brain damage induced by reperfusion injury [16]. Our results were also in agreement with this change. OVX rats which were induced ischemic-reperfusion injury by the temporary occlusion of middle cerebral artery showed the elevation of MDA levels, an index of oxidative stress status, in the cortex area. Interestingly, this change was attenuated by *M. citrifolia* extract at a dose of 50 mg/kg BW. However, no significant changes in the activities of main scavenger enzymes such as SOD, CAT, and GSH-Px enzymes were observed. In addition, the attenuation of cerebral infarction in the cortex was observed in OVX subjected to MCAO and received *M. citrifolia* at all dosage range used in this study. These findings suggested that in addition to oxidative stress, other factors also contribute a role in the brain damage and infarction. Moreover, beyond the main scavenger enzymes mentioned earlier, other factors such as nonenzymatic antioxidant system in the cortex [17] must also play a role in the alteration of oxidative stress status. Based on the elevation of MDA level in the cortex, a product of the chain reaction initiated by peroxyl radicals [18, 19], we did suggest that ischemic-reperfusion injury at the right middle cerebral artery in OVX rats enhanced peroxyl radicals, and this change was mitigated by all doses of *M. citrifolia* used in this study. The possible underlying mechanism might occur via the decreased formation of peroxyl radicals or the increased buffering capacity via nonenzymatic antioxidant system such as glutathione and via other scavenger enzymes such as heme oxygenase-1 and glutathione transferase [18, 19]. It was found that the medium and high doses of extract decreased brain infarction volume, but only the high dose of extract decreased MDA level in the cortex. These data pointed out that other factors might also contribute roles in the reduction of brain infarction volume. Based on an anti-inflammation effect of *M. citrifolia* leaf extract together with the role of inflammation in the brain infarction and dysfunction [20, 21], we suggested that inflammation might also contribute roles in the reduction of brain infarction volume. However, this required further experimental studies to confirm the role of inflammation in the improved brain infarction and brain dysfunction.

It has been reported that ERK signal transduction pathway is stimulated by various stress stimuli [22] such as oxidative stress and amyloid [23, 24], and the upregulation of ERK signal plays an important role in neurodegeneration [24]. In addition to ERK signal transduction pathway, NOS also contributes a role in the induction of neuronal injury in brain ischemia especially in early and late stages of ischemic stroke [25]. Aforementioned changes were in agreement with our data which demonstrated that an ischemic-reperfusion injury induced the activation of ERK signal transduction pathway in the cerebral cortex. This change was attenuated by medium and high doses of *M. citrifolia* extract. However, both genistein and all doses of *M. citrifolia* used in this study failed to modulate the effect of ischemic-reperfusion injury on NOS in the cerebral cortex of OVX rats.

In addition to the neuroprotective effect, *M. citrifolia* also exerted the positive modulation effect on bone dynamic. *M. citrifolia* could increase osteoblast density together with the elevation of serum calcium level but decreased osteoclast density. Although the serum calcium level can occur via both the increased calcium absorption and the increased bone resorption, the current data pointed out that the elevation of serum calcium level occurred as the result of increased calcium absorption derived from a high calcium resource such as *M. citrifolia* leaves based on the decreased osteoclast density. The increase in both serum calcium and osteoblast density together with the elevation of osteocalcin and alkaline phosphatase gave rise to the increase in bone mineralization during bone formation [26–28]. In this study, the antiosteoporotic effect of *M. citrifolia* might be partly due to the increased calcium supplement and the increased osteoblast density giving rise to the enhanced bone formation. The relationship between the alterations of osteoblast density and serum osteocalcin and the relationship between the alterations of osteoblast density and serum alkaline phosphatase failed to show a tight association manner in this study, because the regulation of osteoblast was under multifactors. Other factors such as hormones [29], adipocyte-secreting factors [30], and growth factors [31] also played roles in the regulation of bone formation.

No dose-dependent manner was observed, because the extract used in this study was the crude extract which contained numerous constituents, so the inactive constituents can produce the masking effects on the effect of active constituent. In addition, the observed parameters are under the influences of multifactors, so no simple linear relationship was observed. Our data suggested that the high doses of extract appeared to produce more benefit and warrant further investigations. The explorations concerning the precise underlying mechanisms are still essential before moving forward to clinical trial research.

## 5. Conclusion

The current results suggested that *M. citrifolia* could attenuate brain damage and brain dysfunctions induced by reperfusion injury in OVX rats. The possible mechanism might occur partly via the improvement of oxidative stress status. Other mechanisms such as the decreased ERK1/2 signal transduction might also play the role. In addition, the extract also demonstrated an antiosteoporotic effect by enhancing osteoblast density and the serum levels of calcium, osteocalcin, and alkaline phosphatase leading to the increase in bone formation and mineralization but decreasing osteoclast density in OVX rats. Therefore, our results suggested that *M. citrifolia* is the potential protectant against cerebral ischemia and osteoporosis in menopausal condition.

## Figures and Tables

**Table 1:** Effect of *M. citrifolia* leaf extract on the neurological score of ovariectomized rats which were subjected to a 90-minute ischemic-reperfusion injury at the right middle cerebral artery at 24 hours after the induction of ischemic-reperfusion injury (*N* = 6/group). ^a^*p* value < 0.05, ^aaa^*p* value < 0.05, and *p <* 0.001, respectively, compared to OVX + sham operation. ^∗^*p* value < 0.05, ^∗∗^*p* value < 0.01, and ^∗∗∗^*p* value < 0.001, respectively, compared to OVX + MCAO.

Parameter	Spontaneous activity	Symmetry in the movement of the four limbs	Forepaw outstretching	Climbing	Response to vibrissal touch	Body proprioception
OVX + sham operation	3.00 ± 0.00	3.00 ± 0.00	3.00 ± 0.00	3.00 ± 0.00	3.00 ± 0.00	3.00 ± 0.00
OVX + vehicle + MCAO	2.33 ± 0.67	2.00 ± 0.17^a^	1.00 ± 0.58^aaa^	2.00 ± 0.00^a^	1.33 ± 0.33^aaa^	1.00 ± 0.00^aaa^
OVX + positive + MCAO	2.00 ± 0.58	1.67 ± 0.00	2.67 ± 0.33	2.00 ± 0.58	2.67 ± 0.33	2.33 ± 0.67^∗^
OVX *+ M. citrifolia* (2 mg/kg BW) + MCAO	2.67 ± 0.33	2.00 ± 0.33	2.67 ± 0.33	2.67 ± 0.33	3.00 ± 0.00^∗∗^	3.00 ± 0.00^∗∗∗^
OVX + *M. citrifolia* (10 mg/kg BW) + MCAO	1.75 ± 0.75	2.00 ± 0.71	2.00 ± 0.71	2.50 ± 0.29	2.25 ± 0.48	2.50 ± 0.29^∗∗^
OVX + *M. citrifolia* (50 mg/kg BW) + MCAO	2.75 ± 0.25	2.50 ± 0.29	2.75 ± 0.25^∗^	2.50 ± 0.29	2.75 ± 0.25^∗^	2.75 ± 0.25^∗∗^

**Table 2:** Effect of *M. citrifolia* leaf extract on the oxidative stress status including the level of malondialdehyde (MDA) and the activities of main scavenger enzymes such as superoxide dismutase (SOD), catalase (CAT), and glutathione peroxidase (GPx) in the cerebral cortex of OVX rats which were subjected to MCAO (*N* = 6/group). ^aaa^*p* value < 0.001 compared to OVX + sham operation group. ^∗^*p* value < 0.05 and ^∗∗∗^*p* value < 0.001, respectively, compared to OVX + MCAO group.

Treatment	MDA (nm/mg protein)	SOD (unit/mg protein)	CAT (unit/mg protein)	GPx (unit/mg protein)
OVX + sham operation	0.97 ± 0.16	25.13 ± 8.20	25.45 ± 8.88	3.45 ± 0.92
OVX + MCAO	2.96 ± 0.30^aaa^	14.27 ± 2.65	18.03 ± 5.98	1.96 ± 0.29
OVX + MCAO + genistein	2.79 ± 0.06	13.86 ± 1.14	6.42 ± 0.63	2.68 ± 0.76
OVX + MCAO + *M. citrifolia* (2 mg/kg)	2.51 ± 0.72	34.65 ± 6.83^∗^	22.26 ± 0.80	6.00 ± 1.03^∗^
OVX + MCAO + *M. citrifolia* (10 mg/kg)	2.58 ± 0.35	21.85 ± 3.14	25.54 ± 5.83	4.24 ± 0.24
OVX + MCAO + *M. citrifolia* (50 mg/kg)	0.24 ± 0.05^∗∗∗^	25.22 ± 5.54	22.82 ± 4.30	4.76 ± 0.11
